# Elevated plasma D-dimer levels in dermatomyositis patients with cutaneous manifestations

**DOI:** 10.1038/s41598-018-38108-y

**Published:** 2019-02-05

**Authors:** Koji Habe, Hideo Wada, Ayaka Higashiyama, Tomoko Akeda, Kenshiro Tsuda, Ryoko Mori, Masato Kakeda, Keiichi Yamanaka, Hitoshi Mizutani

**Affiliations:** 1 Department of Dermatology, Mie University Graduate School of Medicine, Mie Tsu, Japan; 2 Department of Molecular and Laboratory Medicine, Mie University Graduate School of Medicine, Mie Tsu, Japan

## Abstract

To explore the influence of dermatomyositis (DM)-specific cutaneous manifestations (scm) on systemic coagulation and fibrinolysis, we retrospectively studied plasma D-dimer levels with/without venous thromboembolism (VTE), malignancy, infection or other connective tissue diseases (CTDs) and scm. One hundred fifty patients with DM were retrospectively investigated using medical records regarding scm, VTE, malignancy, infection, other CTDs, laboratory data and systemic corticosteroid therapy. All DM patients were categorized as follows: group 1, without scm, VTE, infection, malignancy or other accompanying CTDs; group 2, with scm only; and group 3, with VTE, infection, malignancy and other accompanying CTDs but without scm. The D-dimer plasma levels were significantly increased in group 3 compared with healthy subjects and those in groups 1 and 2 (*p* < 0.001). The D-dimer plasma level in group 2 was significantly increased compared with healthy subjects and those in group 1 (*p* < 0.001). Increased D-dimer plasma levels were detected in DM patients with scm without detectable VTE, malignancy, infection or accompanying CTDs. In addition to the known risk factors for increased plasma D-dimer levels in DM patients, including VTE, malignancy, infection and other accompanying autoimmune diseases, the presence of cutaneous manifestations should be considered as a new clinical risk factor.

## Introduction

Venous thromboembolism (VTE) includes deep venous thrombosis (DVT) and pulmonary embolism (PE) and indicates the presence of a risk for emergency and potentially fatal disease^1^. Patients with dermatomyositis (DM) have an increased risk of VTE^2–6^, and the corresponding multivariable hazard ratio for VTE was 8.39 (95% confidence interval [CI]: 3.04–23.14) compared with subjects without DM^7^. DVT is occasionally asymptomatic^8^, and most cases with symptomatic PE have additional asymptomatic DVTs^9^. Therefore, the proper evaluation of accompanying VTE in DM is important.

A close relationship between inflammation and coagulation is well recognized^10,11^. On the other hand, some severe skin inflammatory diseases cause systemic diseases, including cardiovascular disease^12^.

DM is an autoimmune inflammatory disease, with characteristic cutaneous manifestations and inflammatory myopathy. Little is known about the influence of these cutaneous manifestations on systemic coagulation and fibrinolysis systems in DM.

## Methods

### Patients

A retrospective cohort study was performed for all DM patients with data on D-dimer plasma levels who visited the clinic of Dermatology and/or Hematology at Mie University Hospital in Japan from January 1, 2010 to April 30, 2017. DM was diagnosed based on the Classification by Bohan and Peter^13,14^. Clinically amyopathic DM in this study included patients with amyopathic DM or hypomyopathic DM^15,16^. The medical records of 150 DM patients were reviewed, and the records were followed-up from the first diagnosis or first appointment until the end of the study or death. The following patients were excluded: (i) patients receiving preceding anticoagulation therapy or chemotherapy; (ii) patients with other known blood coagulation disorders; and (iii) patients within three months of major surgical therapies, cancer chemotherapy or trauma.

### Methods

VTE was diagnosed based on clinical manifestations and was confirmed by appropriate physical and radiological examinations, including a whole-leg compression ultrasound examination, ventilation-perfusion scan and/or contrast computed tomography (CT).

The malignancy workup included a complete physical examination, chest radiography, whole-body CT, oesophagogastroduodenoscopy, colonoscopy, mammogram, Papnicolaou smear, trans-vaginal ultrasound examination, cystoscopy, bone marrow aspirate and organ biopsy.

Infection was diagnosed based on physical examinations, radiological examinations (chest radiography, head/neck/chest/abdomen/pelvic CT) and clinical laboratory examinations, including a complete blood cell count; plasma levels of C-reactive protein, lactate dehydrogenase, glutamic oxaloacetic transaminase and glutamic pyruvate transaminase; cytomegalovirus antigenaemia assay; serum beta-D-glucan; procalcitonin; urine qualitative measurement and urine sediment examination.

DM patients without VTE, infection or malignancy for three months before and after D-dimer testing were included in the study.

The presence of cutaneous manifestations of DM was evaluated clinically by well-trained dermatologists and histologically by pathologists. Cutaneous manifestations included heliotrope rash, Gottron’s papule, Gottron’s sign, facial erythema, periungual changes, inverse Gottron’s papules (Sign), mechanic’s hands, V-neck sign, shawl sign, flagellate erythema, purpura, calcinosis cutis, skin ulcers and erythroderma^17^.

### Measurement of plasma D-dimer

Plasma D-dimer levels were measured via a latex agglutination method using LPIA-ACE D-Dimer II (LSI Medience Corporation, Tokyo, Japan). The normal range of D-dimer levels by the kit used in the present study was less than 1.0 μg/mL, which was established based on the evaluation of 70 healthy subjects.

When the D-dimer levels were measured more than twice in the same patient, we adopted the D-dimer test results that were obtained on the day closest to when the following events were identified: presence/absence of VTE, infection and malignancy. The D-dimer data obtained shortly after major surgery or trauma were omitted.

The Human Ethics Review Committee of Mie University School of Medicine approved this study protocol (approve number: 1728), and informed consent was obtained from all of the patients. This study was performed in accordance with the principles of the Declaration of Helsinki.

### Statistical analyses

The results were expressed as the medians (Me) (25th–75th percentiles [IQR]). Differences between the groups were analysed using the Mann-Whitney U test. *p*-values < 0.05 were considered statistically significant. The significance of frequency was examined using the chi-squared test. All reported *p*-values are two-sided. All of the statistical analyses were performed using Graphpad Prism software (GraphPad Software, Inc., San Diego, CA, USA).

## Results

The DM patients in this study are listed in Table 1. The final samples included 150 DM patients (112 women, 38 men; mean [standard deviation] age, 60.0 [14.2] years). The median follow-up time of this cohort was 10.0 years (range 0.5–25.0 years), with a total follow-up of 1645.5 person-years. In this study, plasma D-dimer levels were measured when cutaneous manifestation disappeared following treatment in 48% (72/150) of DM patients. VTE (n = 11) included deep vein thrombosis (DVT; n = 8), DVT and pulmonary embolism (PE) (n = 2) and PE (n = 1). Patients with VTE taking intravenous immunoglobulin or oral contraceptive pills were excluded. Cases in whom VTE developed after surgery, long-term recumbence, trauma or an airplane flight were excluded. The calculated incidence rate of VTE in DM patients in this study was 6.68 per 1,000 person-years. Malignancy (n = 18) included breast cancer (n = 6), lung cancer (n = 4), colorectal cancer (n = 3), ovarian cancer (n = 2), gastric cancer (n = 1), bladder cancer (n = 1) and gastric cancer and colorectal cancer (n = 1). Patients in whom malignancy had been completely cured were excluded. Infectious diseases (n = 23) included pneumocystis pneumonia (PCP) (n = 3), cytomegalovirus (CMV) infection and PCP (n = 3), bacterial pneumonia (n = 3), CMV infection (n = 2), herpes zoster (n = 2), phlegmone (n = 2), bacteraemia (n = 1), fungaemia (n = 1), viral enteritis (n = 1), cholecystitis and bacteraemia (n = 1), urinary tract infection (n = 1), subcutaneous abscess (n = 1), lung tuberculosis (n = 1) and nontuberculous mycobacterial pulmonary infection (n = 1). Overlapping CTDs (n = 26) included rheumatoid arthritis (RA; n = 11), systemic sclerosis (SSc; n = 8), Sjogren’s syndrome (SS; n = 3), SSc and RA (n = 2) and SSc and SS (n = 2).

**Table 1:** Subjects’ characteristics.

all DM: n = 150	
age (mean ± SD) (years)	60.0 ± 14.2
Female	112 (75%)
Male	38 (25%)
Classical DM	112 (75%)
Clinically amyopathic DM	38 (25%)
without cutaneous manifestation	72 (48%)
with cutaneous manifestation	78 (52%)
without VTE	139 (93%)
with VTE	11 (7%)
without malignancy	132 (88%)
with malignancy	18 (12%)
without infection	127 (85%)
with infection	23 (15%)
without other CTD	124 (83%)
with other CTD	26 (17%)

DM, dermatomyositis; SD, standard deviation; VTE, venous thromboembolism; CTD, connective tissue disease.

Plasma D-dimer and lactate dehydrogenase (LDH) levels were significantly increased in DM patients with VTE compared with those without VTE. The duration of systemic corticosteroid therapy was longer in DM patients without VTE compared with those with VTE (Me 0.71 years [IQR 0.04–8.5] vs. Me 0.28 years [IQR 0.08–5.2]; *p* = 0.52) (Table 2).

**Table 2:** All DM patients with and without VTE.

	all DM patients: n = 150		*P*-value
	without VTE: n = 139	with VTE: n = 11	
malignancy	n = 17	n = 1	0.758
infection	n = 22	n = 1	0.551
other CTD	n = 23	n = 3	0.366
cutaneous manifestations	n = 74	n = 4	0.281
age (years)	59.0 (51.0, 70.0)	68.0 (56.5, 76.5)	0.093
D-dimer (μg/mL)	1.28 (0.52, 3.66)	4.24 (2.15, 13.76)	<0.001
CPK (IU/L)	53 (29, 130)	57 (22, 109)	0.519
KL-6 (U/ml)	339 (243, 673)	222 (203, 240)	0.159
PLT (×10^4^/μl)	22.3 (18.0, 29.6)	22.9 (14.2, 26.7)	0.310
PT (s)	11.2 (10.5, 11.7)	11.1 (10.5, 14.1)	0.665
APTT (s)	28.1 (25.4, 29.7)	25.8 (22.5, 30.0)	0.183
CRP (mg/dl)	0.15 (0.04, 0.49)	0.43 (0.03, 1.73)	0.272
LDH (IU/l)	244 (209, 327)	306 (252, 438)	0.032
dose of steroid (prednisolone, mg)	13.0 (5.0, 30.0)	13.0 (9.0, 35.0)	0.632
duration of steroid (years)	0.71 (0.04, 8.5)	0.28 (0.08, 5.2)	0.520
total dose of steroid (prednisolone, mg)	8610 (735, 28695)	6283 (0, 100385)	0.653

DM, dermatomyositis; VTE, venous thromboembolism; CTD, connective tissue disease; CPK, creatine phosphokinase; PLT, platelet; PT, prothrombin time; APTT, activated partial thromboplastin time; CRP, C-reactive protein; LDH, lactate dehydrogenase

Normal values: D-dimer, <1.00; CPK, 59–248; KL-6, 105–435; PLT, 15.8–34.8; PT, 9.8–12.1; APTT, ≤37; CRP, ≤0.14; LDH, 124–222.

When all DM patients were divided into two groups according to the normal range of D-dimer levels (<1.0 μg/mL), the incidence of VTE, malignancy, infection and cutaneous manifestations were significantly increased in DM patients with high D-dimer levels (≥1 μg/mL; HDM) compared with those with normal D-dimer levels (<1 μg/mL; LDM) (*p* = 0.016, 0.007, 0.029 and <0.001, respectively). Age, C-reactive protein and LDH levels were significantly increased in the HDM group compared with the LDM group (*p* < 0.001). The platelet (PLT) count was significantly reduced in the HDM group compared with the LDM group (*p* = 0.034), and activated partial thromboplastin time (APTT) was significantly reduced in the HDM group compared with the LDM group (*p* = 0.021). The dose of systemic corticosteroid therapy in the HDM group was increased compared with that in the LDM group, but the duration of corticosteroid therapy in the HDM group was reduced compared with the LDM group (*p* = 0.002 and 0.014, respectively) (Table 3).

**Table 3:** Comparison of all DM patients between the D-dimer <1 μg/mL group and D-dimer ≥1 μg/mL group.

	All DM patients: n = 150		*P*-value
	D-dimer <1 μg/mL: n = 51	D-dimer ≥1 μg/mL: n = 99	
VTE	0	11	0.016
malignancy	1	17	0.007
infection	3	20	0.029
other CTD	12	14	0.175
cutaneous manifestations	13	65	<0.001
CPK (IU/L)	58.0 (36.0, 104.0)	49.0 (24.5, 150.5)	0.260
KL-6 (U/ml)	311 (222, 609)	361 (244, 725)	0.440
age (years)	56.5 (40.5, 60.3)	65 (53.0, 74.0)	<0.001
PLT (×10^4^/μl)	23.1 (20.4, 30.1)	21.5 (16.7, 29.0)	0.034
PT (s)	11.2 (10.6, 11.8)	11.2 (10.5, 11.7)	0.635
APTT (s)	28.5 (26.7, 31.5)	27.1 (24.8, 29.3)	0.021
CRP (mg/dl)	0.070 (0.020, 0.20)	0.20 (0.060, 1.13)	<0.001
LDH (IU/l)	219 (193, 263)	286 (214, 420)	<0.001
dose of steroid (prednisolone, mg)	7.5 (5.0, 18)	20 (8.0, 30)	0.002
duration of steroid (years)	4.9 (0.16, 11)	0.28 (0.038, 6.5)	0.014
total dose of steroid (prednisolone, mg)	16048 (1146, 34072)	5943 (463, 26277)	0.070

The data are presented as the median (Q1, Q3). DM, dermatomyositis; VTE, venous thromboembolism; CTD, connective tissue disease; CPK, creatine phosphokinase; PLT, platelet; PT, prothrombin time; APTT, activated partial thromboplastin time; CRP, C-reactive protein; LDH, lactate dehydrogenase.

Normal values: D-dimer, <1.00; CPK, 59–248; KL-6, 105–435; PLT, 15.8–34.8; PT, 9.8–12.1; APTT, ≤37; CRP, ≤0.14; LDH, 124–222.

In DM patients without VTE, malignancy, infection or other CTD, the plasma levels of D-dimer and LDH were significantly increased in patients with cutaneous manifestations compared with those without cutaneous manifestations (*p* < 0.001 and 0.004, respectively). The duration and total dose of corticosteroid therapy were significantly reduced in patients with cutaneous manifestations compared with those without cutaneous manifestations (*p* < 0.001) (Table 4). Interestingly, 83% (34/41) of DM patients with cutaneous manifestations exhibited increased plasma D-dimer levels (Me [IQR]: 1.56 [0.93–3.48] μg/mL; normal <1 μg/mL); however, this proportion was only 31% (14/45) in patients without cutaneous manifestations (Table 4).

**Table 4:** DM patients with no VTE, malignancy, infection or other CTD.

	DM patients without VTE, malignancy, infection or other CTD: n = 86		*P*-value
	without cutaneous manifestations: n = 45	with cutaneous manifestations: n = 41	
D-dimer (μg/mL)	0.50 (0.26, 1.07)	1.56 (0.93, 3.48)	<0.001
PLT (×10^4^/μl)	22.4 (18.7, 29.8)	20.4 (16.8, 29.3)	0.242
PT (s)	11.0 (10.5, 11.6)	11.4 (10.6, 11.9)	0.092
APTT (s)	28.0 (26.1, 29.0)	28.5 (26.1, 31.2)	0.363
CPK (IU/L)	53 (36, 90)	65 (25, 305)	0.397
KL-6 (U/ml)	301 (232, 483)	351 (223, 749)	0.494
age (years)	58 (51, 63)	58 (46, 73)	0.727
CRP (mg/dl)	0.08 (0.02, 0.27)	0.09 (0.03, 020)	0.913
LDH (IU/l)	217 (201, 255)	261 (211, 371)	0.004
dose of steroid (prednisolone, mg)	9.0 (6.3, 17.5)	20.0 (0, 37.5)	0.231
vduration of steroid (years)	8.5 (1.7, 14)	0.08 (0, 5.3)	<0.001
total dose of steroid (prednisolone, mg)	24000 (10725, 55335)	1093 (0, 14625)	<0.001

The data are presented as the median (Q1, Q3). DM, dermatomyositis; VTE, venous thromboembolism; CTD, connective tissue disease; PLT, Platelet; PT, prothrombin time; APTT, activated partial thromboplastin time; CPK, creatine phosphokinase; CRP, C-reactive protein; LDH, lactate dehydrogenase.

Normal values: D-dimer, <1.00; PLT, 15.8–34.8; PT, 9.8–12.1; APTT, ≤37; CPK, 59–248; KL-6, 105–435; CRP, ≤0.14; LDH, 124–222.

**Figure 1:**
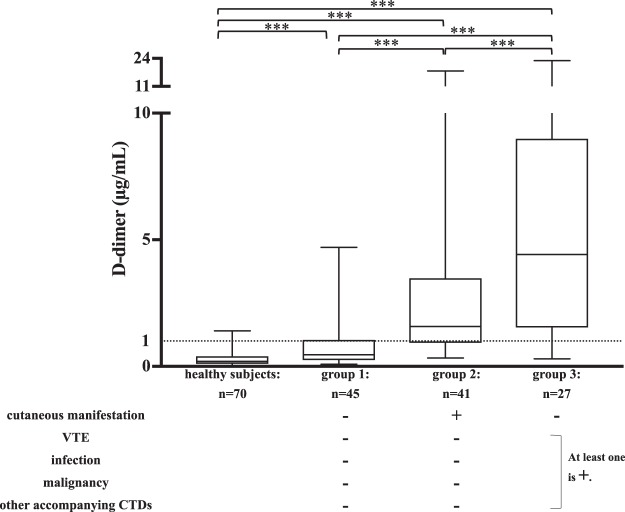
Plasma D-dimer levels in DM patients. ***p < 0.001 (Mann-Whitney U test). DM, dermatomyositis; VTE, venous thromboembolism; CTD, connective tissue disease. Normal values: D-dimer, <1.00.

To further analyse the effects of various clinical manifestations on plasma D-dimer levels, patients were categorized into the following three groups: group 1, without cutaneous manifestations, VTE, infection, malignancy or other accompanying CTDs; group 2, with cutaneous manifestations only; and group 3, with VTE, infection, malignancy and other accompanying CTDs but without cutaneous manifestations. D-dimer plasma levels in the three groups were significantly increased compared with healthy subjects (Me [IQR]; 0.28 [0.10–0.40] μg/mL) (*p* < 0.001). D-dimer plasma levels were significantly increased in group 2 (Me [IQR]; 1.56 [0.93–3.48] μg/mL) compared with group 1 (Me [IQR]; 0.50 [0.26–1.07] μg/mL) (*p* < 0.001). D-dimer plasma levels were also significantly increased in group 3 (Me [IQR]; 4.41 [1.54–9.00] μg/mL) compared with groups 1 and 2 (*p* < 0.001). No significant differences in age or sex were noted between groups (Fig. 1).

No specific skin manifestations were related to increased plasma D-dimer levels (Table 5).

**Table 5:** DM patients with cutaneous manifestations but without VTE, malignancy, infection or other CTD.

	DM patients with cutaneous manifestations but without VTE, malignancy, infection or other CTD: n = 41		*P*-value
	D-dimer <1 μg/mL: n = 7	D-dimer ≥1 μg/mL: n = 34	
heliotrope rash	4	16	0.627
Gottron’s papule	4	19	0.951
Gottron’s sign	5	24	0.700
facial erythema	3	11	0.594
periungual changes	7	25	0.123
inverse Gottron’s papules (Sign)	1	7	0.702
mechanic’s hands	2	6	0.501
V-neck sign	1	5	0.853
shawl sign	0	4	0.569
flagellate erythema	0	2	1.000
purpura	1	6	1.000
calcinosis cutis	0	1	1.000
skin ulcers	0	8	0.179
erythroderma	0	1	1.000

DM, dermatomyositis; VTE, venous thromboembolism; CTD, connective tissue disease.

## Discussion

VTE occasionally requires emergency care and is a potentially fatal disease. Fortunately, the incidence of VTE in the general population is relatively low at approximately 1.17 per 1,000 person-years in a cohort of Olmsted County^18^. An increased risk for VTE has been reported in patients with DM^3–7^. Surprisingly, the incidence rate of VTE in DM patients in this study was relatively high at 6.68 per 1,000 person-years. However, the causative factors for VTE in DM remain unclear. Immobility increases the risk of VTE, but the serum CPK levels of the patients with VTE in this study were not increased. Thus, immobility due to severe myositis is not suspected for VTE in DM. Serum LDH levels were increased in DM patients with VTE compared with those without VTE. LDH isozyme in the lung is a component of LDH, and LDH serum levels are increased in PE^19,20^. In this study, VTE (n = 11) was noted in 3 PE patients, thus influencing this result. Systemic corticosteroid therapy activates coagulation cascades^21^ and increases the risk of VTE^22^. Indeed, in this study, APTT was significantly reduced in the HDM group, which was prescribed greater doses of steroids, compared with the LDM group. The difference seemed minimal, but the steroid dose potentially influenced this difference. However, no marked differences in the dose, duration or total dose of systemic corticosteroids were noted between patients with and without VTE in the present study. Thus, neither the severity of myositis nor the therapeutic use of corticosteroids increased the risk of VTE in DM patients in this study. The PLT count was significantly reduced in the HDM group compared with the LDM group. This difference was minimal and might have been affected by the number of infection and malignancy patients in the HDM group.

D-dimers are the products of fibrin hydrolysis, and increased D-dimer levels indicate the presence and digestion of stable fibrin and the activation of the coagulation and fibrinolysis cascades^23^. The evaluation of plasma D-dimer levels is accepted as a clinical diagnostic test for the presence of VTE^24^. VTE is a potentially fatal disease, and surveillance of VTE using clinical laboratory tests, including the D-dimer test, is useful for the early detection of VTE of DM. D-dimer levels are also increased in various accompanying diseases, and the careful evaluation of plasma D-dimer levels in cases without clinically obvious thromboembolic disease is required (Supplementary Figure). DM patients are commonly complicated by malignancy, infection and/or other CTDs or RA. Previous studies have demonstrated increased plasma D-dimer levels in patients without demonstrable thrombosis but with malignancy^25^, infection^26^ and other autoimmune diseases^27^.

A close relationship between inflammation and coagulation is well recognized^10,11^. Various inflammatory mediators activate the coagulation systems, thus downregulating natural anticoagulant mechanisms^28^. Increased plasma D-dimer levels are not limited to thrombotic diseases and have been reported in some skin diseases, including urticaria^29,30^, angioedema^31^, bullous pemphigoid^32^, atopic dermatitis^33^ and cutaneous drug reactions^33^. These diseases have different pathogenic mechanisms, and the existence of multiple different mechanisms underlying increased plasma D-dimer is suspected. Therefore, inflammatory reactions of the cutaneous manifestation itself might be involved in the increased plasma D-dimer levels. Microvascular endothelial injury is a characteristic pathogenic mechanism of DM. The vascular endothelium plays important roles in haemostasis and thrombosis, and an inflammatory mediator-stimulated endothelium initiates coagulation system activation. In this study, plasma D-dimer levels were increased in DM patients with skin manifestations, possibly due to endothelial cell injury associated with DM.

**Figure 2:**
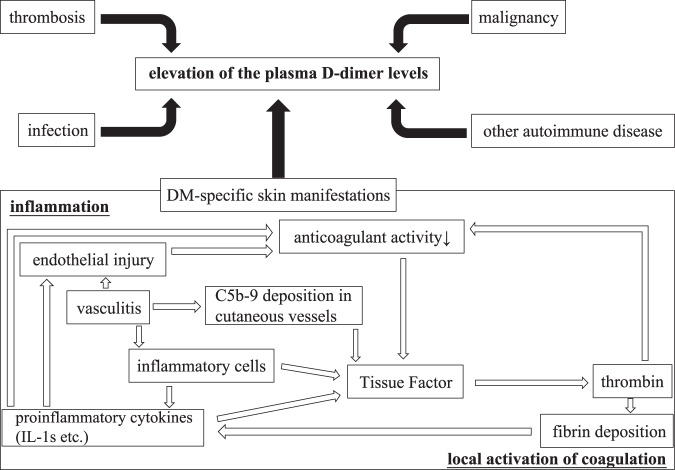
Pathogenesis of the increased plasma D-dimer levels in DM patients. DM, dermatomyositis.

DM-specific skin manifestations represent clinically obvious small-vessel vasculitis and capillary thrombosis, including Gottron’s sign and periungual changes (nail fold thrombosis). These lesions also exhibit histopathological changes of cutaneous vasculitis^34,35^, vascular fibrin deposition^36,37^ and C5b-9 deposition in cutaneous vessels^36^ as well as endothelial injury^36^. Vascular fibrin deposition was demonstrated in 65% of skin biopsies of DM patients, (93% in amyopathic DM), C5b-9 deposition in 100% (100% in amyopathic DM) and endothelial injury in 78% (100% in amyopathic DM)^36^. Fibrin is hydrolysed to produce D-dimers, and C5b-9 is associated with coagulation^38^. Increases in plasma D-dimer levels have been reported in various autoimmune vasculitis conditions, including antineutrophil cytoplasmic antibody-associated vasculitis^39^, cutaneous polyarteritis nodosa^29^, Takayasu’s arteritis^40^, eosinophilic granulomatosis with polyangiitis^41^ and IgA vasculitis^42^. Thus, vasculitis induced by skin manifestations is suspected to be pathogenic for plasma D-dimer elevation in DM. Regarding the association between inflammatory cytokines and DM, inflammatory cytokine expression (IL-1α, IL-1β and TNF-α) is increased in the muscular lesions of DM^43^. The involvement of IL-1s in the pathogenesis of skin manifestations in DM was proven based on the clinical effects of IL-1 antagonist when administered for the treatment of DM-associated skin ulcers^44^. Inflammatory cytokines affect tissue factor by playing a central role in the initiation of inflammation-induced coagulation and the regulation of physiologic anticoagulation^11,45^ (Fig. 2). In addition, a positive association has been reported between inflammatory mediators and VTE recurrence^46^.

Within DM-specific skin manifestations, as described earlier, coagulation is thought to be locally activated according to local inflammation associated with the development of DM-specific skin manifestations (Fig. 2). In this study, increased plasma D-dimer levels were associated with DM-specific skin manifestations, even if there was no thrombosis, infection, malignancy or other autoimmune disease (Fig. 1). From this, strong inflammation within DM-specific skin manifestations is thought to first induce local activation of coagulation, which potentially leads to the systemic activation of coagulation. When considering other pathologies of increased D-dimer plasma levels in DM patients with DM-specific cutaneous manifestations, it is also possible that intravascular inflammation influences the systemic activation of coagulation. However, in this study, CRP plasma levels were not significantly increased in DM patients with DM-specific cutaneous manifestations compared with those of DM patients without DM-specific cutaneous manifestations (Table 4). Therefore, inflammation within DM-specific skin manifestations likely influences the systemic activation of coagulation.

LDH is present in various organs, including the skin, lung and others. We thus considered the effect of LDH on pulmonary infarction (Table 2) and skin (Table 4). In Table 3, we also considered the effect from various organs affected by malignancy or infection as well as pulmonary infarction and skin. Clinically, LDH is not a specific test for the assessment of suspected DVT or the examination of coagulation compared with D-dimer levels. In DM, a clinically important association is noted between increased plasma D-dimer levels and DM-specific skin manifestations, as our current evidence suggests. This finding suggests that the plasma D-dimer levels in DM patients exhibit clinical importance similar to that noted in SLE^47^.

The limitations associated with our study are its retrospective nature and the limited number of patients involved. In addition, we cannot completely exclude the possible presence of micro VTE in patients with increased plasma D-dimer levels, which is undetectable, even when using various imaging examinations.

However, the D-dimer levels in 7 (17%) of 41 DM patients with skin manifestations were within the normal range in this study (Table 4). Therefore, the presence of cutaneous manifestations in DM patients does not necessarily indicate increased plasma D-dimer levels. This finding might be due to the time of D-dimer sampling during the clinical course of DM and the heterogeneity of skin manifestations in DM. Unfortunately, we were unable to detect a specific DM skin lesion relevant to D-dimer elevation in this study due to the relatively small sample size (Table 5). A long lag time is occasionally present between D-dimer elevation and PE and DVT development^48^. Further studies are required to determine specific skin manifestations and the ideal sampling time for the evaluation of D-dimer levels for the risk of VTE management.

## Conclusions

In the present study, we identified DM-specific cutaneous manifestations as a new marker for activated coagulation cascade and increased plasma D-dimer levels. The risk of VTE is increased in DM and SLE patients, and we should carefully monitor plasma D-dimer levels, skin manifestations and clinical manifestations of thrombotic events in DM patients.

## Supplementary information

Dataset 1
