# Supplementary material for: Elevated plasma D-dimer levels in dermatomyositis patients with cutaneous manifestations
Source: Sci Rep. 2019 Feb 5;9:1410. doi: 10.1038/s41598-018-38108-y (PMC6363793; doi:10.1038/s41598-018-38108-y)
Supplement: Supplementary file 1 — Dataset 1 [file 41598_2018_38108_MOESM1_ESM.pdf]

Supplementary Information for *Scientific Reports*

Elevated plasma D-dimer levels in dermatomyositis patients with cutaneou manifestations

Koji Habe, MD, PhD; Hideo Wada, MD, PhD; Ayaka Higashiyama, MD; Tomoko Akeda, MD, PhD; Kenshiro Tsuda, MD, PhD; Ryoko Mori, MD; Masato Kakeda, MD, PhD; Keiichi Yamanaka, MD, PhD; Hitoshi Mizutani, MD, PhD

Supplementary Figure: Plasma D-dimer levels in DM patients without cutaneous manifestation

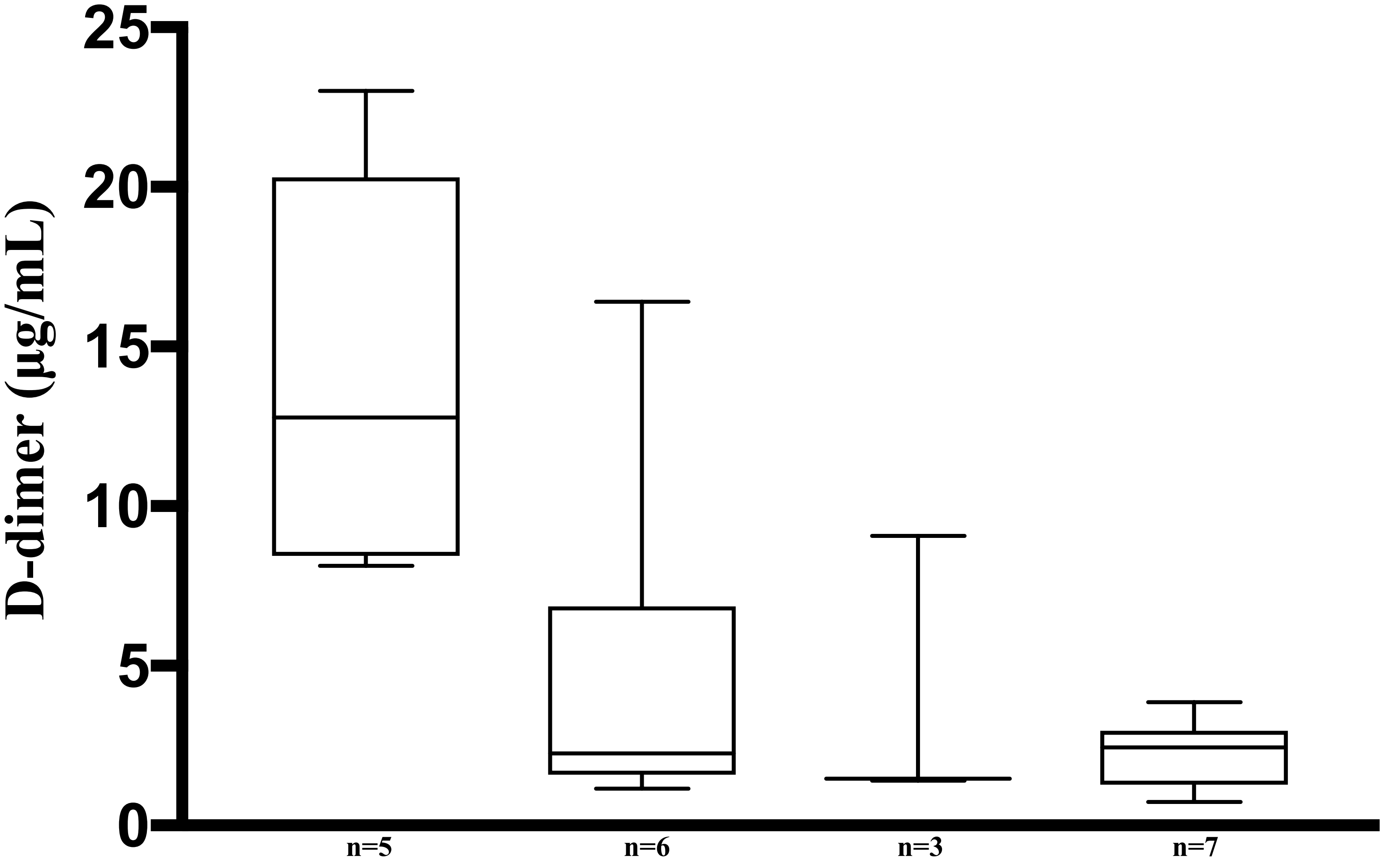

cutaneous manifestation	−	−	−	−
VTE	+	−	−	−
infection	−	+	−	−
malignancy	−	−	+	−
other accompanying CTDs	−	−	−	+

DM, dermatomyositis; VTE, venous thromboembolism; CTD, connective tissue disease.
